# Isothermal Heteroepitaxy of Ge_1–*x*_Sn_*x*_ Structures for Electronic and Photonic Applications

**DOI:** 10.1021/acsaelm.3c00112

**Published:** 2023-04-03

**Authors:** Omar Concepción, Nicolaj B. Søgaard, Jin-Hee Bae, Yuji Yamamoto, Andreas T. Tiedemann, Zoran Ikonic, Giovanni Capellini, Qing-Tai Zhao, Detlev Grützmacher, Dan Buca

**Affiliations:** † Peter Gruenberg Institute 9 (PGI-9), Forschungszentrum Juelich, 52428 Juelich, Germany; ‡ Interdisciplinary Nanoscience Center (iNANO), Aarhus University, 8000 Aarhus C, Denmark; § IHP - Leibniz Institut für innovative Mikroelektronik, Im Technologiepark 25, 15236 Frankfurt (Oder), Germany; ∥ Pollard Institute, School of Electronic and Electrical Engineering, University of Leeds, Leeds LS2 9JT, United Kingdom; ⊥ Dipartimento di Scienze, Università Roma Tre, Viale G. Marconi 446, 00146 Roma, Italy

**Keywords:** GeSn alloy, chemical vapor deposition, isothermal heterostructures, epitaxial growth, optoelectronic applications

## Abstract

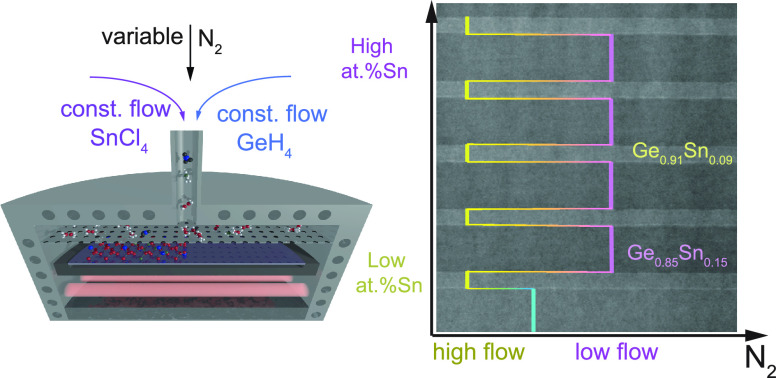

Epitaxy of semiconductor-based quantum well structures is a challenging task since it requires precise control of the deposition at the submonolayer scale. In the case of Ge_1–*x*_Sn_*x*_ alloys, the growth is particularly demanding since the lattice strain and the process temperature greatly impact the composition of the epitaxial layers. In this paper, the realization of high-quality pseudomorphic Ge_1–*x*_Sn_*x*_ layers with Sn content ranging from 6 at. % up to 15 at. % using isothermal processes in an industry-compatible reduced-pressure chemical vapor deposition reactor is presented. The epitaxy of Ge_1–*x*_Sn_*x*_ layers has been optimized for a standard process offering a high Sn concentration at a large process window. By varying the N_2_ carrier gas flow, isothermal heterostructure designs suitable for quantum transport and spintronic devices are obtained.

## Introduction

Group-IV Ge_1–*x*_Sn_*x*_ alloys are attracting ever-growing interest as enablers of the extension of the Si-photonics technological platform toward the near-/mid-infrared region (NIR/MIR) of the electromagnetic spectrum. This comes following the experimental demonstration of a unique property for the group-IV semiconductors, a fundamental direct band gap in Ge_1–*x*_Sn_*x*_ alloys^1^ that led to the realization of optically pumped lasers up to room temperature^2^ and also electrically injected Ge_1–*x*_Sn_*x*_ lasers operating at low temperatures.^3^ Moreover, the previously short-wave infrared range dominated by III-V materials was reached and extended into MIR as demonstrated recently by Ge/Ge_1–*x*_Sn_*x*_ single pixel imagers on Si.^4^

Furthermore, the Ge_1–*x*_Sn_*x*_ material system is making its way into a diversity of research fields, such as nanoelectronics,^5^ thermoelectrics,^6^ spintronics,^7^ and quantum computing.^8^ Due to their strong spin–orbit coupling (SOC), holes in Ge have emerged as one of the most promising spin qubit candidates.^9,10^ To further enhance the SOC strength, materials with higher atomic numbers are preferred due to larger atomic potential variation, which is achieved by the incorporation of Sn atoms into Ge crystals.^11^

Different from laser structures, which typically require thick layers and very large Sn contents, to widely separate the Γ and L- valleys of the conduction band, for electronic transport thin defect-free Ge_1–*x*_Sn_*x*_ layers or quantum wells (QWs) heterostructures are desirable. Such advanced heterostructures, which employ Ge or Si_1–*x*–y_Ge_y_Sn_*x*_ barrier layers to define Ge_1–*x*_Sn_*x*_ QWs, are extremely challenging for the epitaxial growth.^12^ For example, even the simple growth of a stack in which a Ge_1–*x*_Sn_*x*_ layer is deposited on another Ge_1–*y*_Sn_*y*_, *x* < *y* layer (inverse step Sn composition) cannot be easily performed. Indeed, to get a lower Sn content, one typically has to increase the deposition temperature. However, this temperature increase leads to crystallinity degradation of the already grown layer(s) via Sn diffusion or even segregation.^13^ Therefore, the layer with the highest Sn content in a heterostructure defines the maximum growth temperature for the epitaxy of a complex heterostructure.^14−16^ Presently, to circumvent the problems of large lattice mismatch, large built-in compressive strain, and low Sn solubility, high Sn content Ge_1–*x*_Sn_*x*_ layers have been grown on micrometer-thick Ge_1–*x*_Sn_*x*_ buffers where the Sn content is increased gradually or in small steps.^17^ This approach limits the scalability and consequently the applicability of these structures, for example, for metal oxide semiconductor field-effect transistors (MOSFETs).

Two commercially available Ge precursors, germane (GeH_4_) and digermane (Ge_2_H_6_), are commonly used in (Si)GeSn chemical vapor deposition (CVD) reactors, while SnCl_4_ is the precursor of choice for the Sn atoms. The increased reactivity of Ge_2_H_6_ at low growth temperatures and the resulting high growth rates makes it ideal for the epitaxy of thick relaxed layers.^1,18^ However, Ge_2_H_6_ is significantly more expensive than GeH_4_, has limited availability, and the films may be accompanied by the formation of defects.^19−21^ On the other hand, GeH_4_ offers lower growth rates and a narrower window of the growth parameters, like the reactor pressure, gas flow rates, and temperature, which offers high crystallinity Sn-rich Ge_1–*x*_Sn_*x*_ alloys.^22,23^ It then becomes clear that these parameters have a strong impact on the gas phase reactions of different molecules like GeH_4_ and Ge_2_H_6_. The preferred reaction partner for the stable SnCl_4_ molecule on the substrate surface are GeH_*x*_ (*x* = 1,2,3) radicals. The formation of those in the gas phase is heavily impacted by the aforementioned growth parameters. An additional parameter suitable to influence gas phase reactions is the composition of the carrier gas, i.e., mixtures of H_2_ and N_2_.^24^ A high H_2_ concentration reduces the cracking rate of GeH_4_ and Ge_2_H_6_ when compared to the high N_2_ concentration and increases the back-reaction rate from GeH_*x*_-radicals to GeH_4_ molecules. However, it has to be kept in mind that the H_2_ ambient supports the hydrogen passivation of the surface, preventing Ge segregation^25^ as well as surface oxidation.^26^ Most importantly, the molecules prepared by gas phase reactions at the substrate surface are crucial for the subsequent surface kinetics guiding the CVD growth at low temperatures.^27^

In this paper, the epitaxy of thin Ge_1–*x*_Sn_*x*_ alloys using GeH_4_ and SnCl_4_ precursors is revisited, targeting a simple methodology that uses the N_2_ gas flow as the main growth parameter to realize isothermal heterostructures. In particular, the growth of layer sequences, like quantum wells with large variations of Sn concentration in the Ge_1–*x*_Sn_*x*_ heterostructures, is enabled by keeping the process temperature, the reactor pressure, and the flow of the gas precursors constant. In this way, the gas flow rate and the partial pressure of the precursors change, and the gas phase reactions are thus modified with impact on the surface kinetics, allowing precise control of the Sn concentration. The knowledge gained is exploited to realize isothermally epitaxial GeSn-based heterostructures aimed for electronic-, spintronic-, and photonic-device applications.

## Experimental Methods

The Ge_1–*x*_Sn_*x*_ epitaxy was performed in an industry compatible 300 mm/200 mm AIXTRON TRICENT reduced-pressure chemical vapor deposition (RP-CVD) reactor with a showerhead technology that provides a small reactor volume and a uniform gas precursor distribution over the whole wafer. Si (001) wafers (200 mm) were cleaned *ex situ* with HF vapor using an automated cleaning tool, followed by an *in situ* pre-epi bake at about 1000 °C. Two types of Ge/Si substrates were used to reduce the lattice mismatch between Ge_1–*x*_Sn_*x*_ and the Si substrate: (i) a 300 nm-thick Ge buffer layer grown at 450 °C on Si (001) substrates before the Ge_1–*x*_Sn_*x*_ layer deposition (one epitaxy run) and (ii) a previously grown 1.5 μm-thick, high quality, cycle-annealed Ge/Si(001) virtual substrate (VS) (see details in ref (28)).

Germane, GeH_4_ (10% diluted in H_2_), and tin-tetrachloride (SnCl_4_) precursors were employed, while N_2_ was used as carrier gas. Based on previous Ge_1–*x*_Sn_*x*_ growth experience using Ge_2_H_6_,^29^ in agreement with the literature reports,^30^ the precursor flow was scaled and adjusted to obtain mirror-like wafers with high-quality Ge_1–*x*_Sn_*x*_ layers. Using GeH_4_ instead of Ge_2_H_6_, the amount of GeH_3_ reactive molecules available in the reactor is reduced by half for the same gas flow, and considering the activation energy of 1.3 eV for GeH_4_, higher than 0.7 eV for Ge_2_H_6_,^24^ the GeH_4_ gas flow was increased by a factor of 4, in-line with Hartmann et al.’s^30^ findings. The used growth parameters of partial pressure ratio *p*_GeH4_/*p*_SnCl4_ = 1100, reactor pressure of *P*_react_ = 60 mbar, and total gas flow *Q*_total_ = 9000 sccm are further called “reference” growth conditions.

The stoichiometry, thickness, and crystal quality of the epitaxial layers were extracted by fitting the Rutherford backscattering spectra (RBS) taken at random and channeling alignment using a Tandetron accelerator with 1.4 MeV He^+^ ions at a backscattering angle of 170°. The crystal orientation and the strain build-up in the films were determined by X-ray diffraction (XRD) and reciprocal space mapping (RSM), respectively, while the crystal quality was verified by transmission electron microscopy (TEM) imaging. Finally, temperature-dependent photoluminescence (PL) spectra and electronic band structure calculations were performed to address the suitability of the Ge_1–*x*_Sn_*x*_ heterostructures for specific applications.

## Results and Discussion

### Reference Growth Conditions - Temperature Dependence

**Figure 1:**
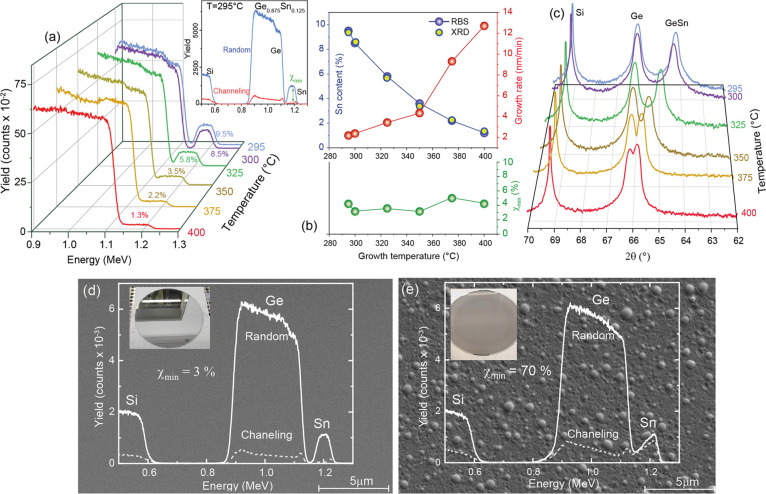
(a) 3D plot of RBS random spectra of Ge_1–*x*_Sn_*x*_ layers grown at different process temperatures. Inset: the full random and channeling spectra of the sample grown at 295 °C. (b) Sn content, growth rate, and the RBS minimum channeling yield as a function of the substrate temperature. (c) Symmetric 2θ-ω XRD spectra along the (004) plane of the same set of samples. SEM and optical images (inset) of samples with (d) mirror-like and (e) Sn segregation surfaces overlapped with the RBS random and channeling spectra.

The classical pathway toward high Sn content layers is the decrease of the process temperature while keeping all other growth parameters constant. A set of wafers has been grown on 300 nm-thick Ge/Si (001) substrates at temperatures ranging between 295 and 400 °C, using the reference growth conditions given above. The layer’s thickness and stoichiometry were obtained by fitting the RBS random spectra as shown in Figure 1a. For clarity, only the energy region between 0.9 and 1.3 MeV, corresponding to the Ge and Sn signals in the Ge_1–*x*_Sn_*x*_ layers is shown, while a full RBS spectrum is exemplified in the inset. The plateaus visible at a backscattered ion energy of ∼1.2 MeV are evidence of uniform depth distribution of the Sn atoms. The Sn concentration increases from about 1 at. % up to 9.5 at. % by decreasing the growth temperature from 400 to 295 °C, as shown in Figure 1b (left scale, top panel). At a lower temperature of 290 °C, the Sn content reaches ∼10 at. % and the wafer presents a mirror-like surface, but isolated Sn segregation occurs. Further temperature decrease leads to a large Sn segregation, as illustrated in the scanning electron microscope (SEM) and optical images (inset) presented in Figure 1d,e, showing a mirror-like and a Sn segregation surface, respectively. The growth rate determined from the thickness extracted from the RBS fitting (Figure 1b) varies strongly with the temperature from ∼13 nm/min at 400 °C to 2 nm/min at 295 °C. This behavior for different process temperatures is in perfect agreement with the literature reports.^30−32^

The crystalline quality of the Ge_1–*x*_Sn_*x*_ alloy is visible from the minimum channeling yield χ_min_ (Figure 1b, bottom panel), defined as the ratio of intensities between channeling and random RBS spectra (Figure 1a) measured directly behind the surface peak. In agreement with previous reports,^33,34^ the low χ_min_ ≤ 6% for all samples indicates the high Ge_1–*x*_Sn_*x*_ single-crystalline quality and high substitutability of the Sn atoms. The opposite is obtained in the samples where Sn segregation occurs, characterized by high values of χ_min_ and nonuniform plateau (Figure 1e).

The results are confirmed by the symmetric XRD spectra taken along the (004) plane (Figure 1c) where the Ge_1–*x*_Sn_*x*_ diffraction peaks systematically shift away from the Ge peak toward lower 2θ angles, as a result of the increasing out-of-plane lattice constant due to higher Sn incorporation and correspondingly increased compressive biaxial strain. From the position of the Ge_1–*x*_Sn_*x*_ peak in the RSMs, and using Vegard’s law with a bowing parameter of 0.04, the Sn content was extracted (yellow symbols in Figure 1b), showing an excellent agreement with RBS fitting. All Ge_1–*x*_Sn_*x*_ layers grown here are fully compressively strained (pseudomorphic growth), evidenced from the same in-plane lattice constant with the Ge buffer layer, which is slightly tensile strained (0.15%) (see Figure S1 in the Supporting Information (SI) file). The build-up of tetragonal elastic strain in the Ge_1–*x*_Sn_*x*_ alloy for different Sn compositions changes from almost a cubic lattice (∼0% strain) at 400 °C to −1.25% compressive biaxial strain for Ge_0.905_Sn_0.095_ layer growth at 295 °C.

### Isothermal Growth Conditions

The typical approaches for tuning the alloy composition^21,22,30,35,36^ at constant process temperature are performed by changing the precursors’ flow or the reactor pressure. Starting from the reference growth conditions at 300 °C, the decrease/increase in the GeH_4_ and SnCl_4_ partial pressures through the direct control of the flux of these precursors results in small changes of the Sn incorporation, giving a maximum Sn concentration of 9.0–9.5 at. %, compared to the 8.5 at. % Sn obtained under the reference growth conditions (Figure S2a,b of the SI). Another way to increase the Sn incorporation is to change the reactor pressure while keeping the total flow and the precursors flow constants as the “reference” values, meaning a constant *p*_GeH4_/*p*_SnCl4_ ratio. The reactor pressure change between 60 and 200 mbar translating into a SnCl_4_ partial pressure between 0.05 (reference growth conditions) and 0.16 Pa results in a maximum Sn incorporation of 10.5 at. % at the highest reactor pressure value (Figure S2c of the SI).

For some electronic devices like nanowire MOSFETs where the nanowire patterning relaxes the strain in the Ge_1–*x*_Sn_*x*_ layer,^5^ an Sn content around 10 at. % might be sufficient, but larger Sn contents are still required to reach a strongly direct band gap for photonic applications like light emitter or lasers. The next growth parameter to study, which may increase the Sn incorporation, is the N_2_ carrier gas flow. Both parameters, increasing the total pressure or decreasing the N_2_ flow rate, have similar effects. The precursor partial pressures are proportionally increased with the factor of pressure increase or N_2_ flow reduction. The gas flow rate decreases, i.e., the retention time of the reactive precursors in the reactor increases for both cases. Consequently, the amount of GeH_*x*_ radicals on the surface will increase as well.

**Figure 2:**
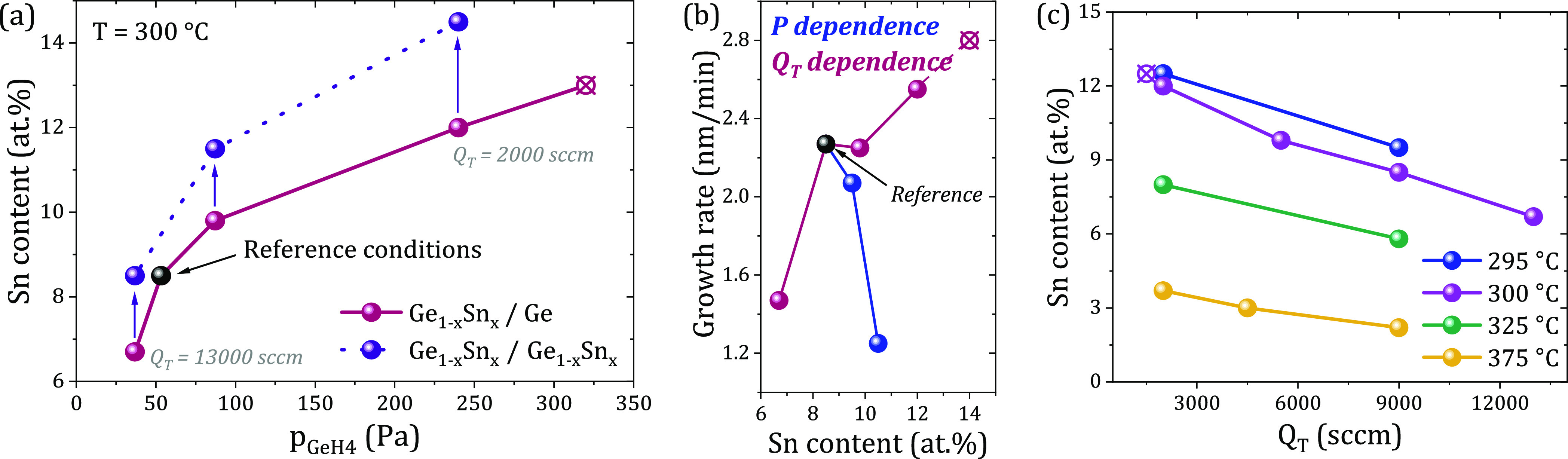
(a) Sn content incorporation at 300 °C as a function of the partial pressure of GeH_4_ for different total N_2_ flows grown onto Ge (pink) and Ge_1–*x*_Sn_*x*_ (purple) buffer layers. The black point represents the “reference” growth conditions, and the cross-white symbols correspond to samples that show Sn segregation on the surface. (b) Growth rate as a function of the Sn content corresponding to changes in the reactor pressure and total N_2_ flow. (c) Summary of the Sn content as a function of total carrier gas flow at different growth temperatures showing similar behavior.

The total gas flow in the reactor, *Q*_T_, (Figure 2a) is adjusted by tuning the N_2_ carrier gas flow at constant total pressure and a ratio *p*_GeH4_/*p*_SnCl4_ = 1100 (“reference” growth condition). In this way, the gas flow rate and the partial pressure of the precursors change, and therefore the gas phase reactions are modified, with an impact on the surface kinetics. Keeping the growth temperature of 300 °C, the decrease of *Q*_T_ from 13,000 to 2000 sccm greatly increases the Sn content from 6.5 to 12 at. %. Since the precursor fluxes have been kept constant, their partial pressures increase by a factor of 6.5. A further decrease in the N_2_ flow leads to a SnCl_4_ partial pressure of 0.3 Pa at which Sn segregation takes place (empty symbol, Figure 2a).

The growth rates corresponding to the pressure and total gas flow variations are shown in Figure 2b. Interestingly, the Ge_1–*x*_Sn_*x*_ growth rate has a completely opposite behavior: while it decreases with the increasing Sn content under pressure variation, the highest growth rate is obtained for the larger Sn incorporation by the N_2_ flow change. Moreover, the same growth rate of ∼2.6–2.8 nm/min is obtained also by changing the SnCl_4_ flow (Figure S2b of SI); however, it offers only ∼9 at. % Sn incorporation compared to 12 at. % Sn in the case of N_2_ flow tuning. These important observations make the N_2_ flow change method very attractive for high Sn content device epitaxy.

To summarize the data at different growth temperatures, the plot in Figure 2c shows the relation between the Sn content in the Ge_1–*x*_Sn_*x*_ alloy and the total gas flow at different process temperatures. It becomes clear that increasing the SnCl_4_ partial pressure through the decrease of the total flow stimulates the Sn incorporation in the Ge lattice regardless of the process temperature. This plot shows that a large stoichiometry tuning in Ge_1–*x*_Sn_*x*_ multilayers can be obtained by isothermal epitaxial growth only via total carrier flow.

The above observation (pink curve in Figure 2a) refers to lattice-matched Ge_1–*x*_Sn_*x*_ epitaxy directly on Ge buffer. However, if under the same growth conditions, the film is grown beyond the critical thickness, the bottom Ge_1–*x*_Sn_*x*_ relaxes, offering a larger lattice constant, reduced surface strain, and increased surface mobility of the adatoms. These lead to a considerably larger Sn content incorporation into the subsequent Ge_1–*x*_Sn_*x*_ epitaxial layer^37−39^ (purple curve in Figure 2a). Under a constant total flow of 2000 sccm and increasing the growth time, it is possible to increase the Sn concentration from 11.5 to 14.5 at. %. Such layers were successfully used as optical active media for Ge_1–*x*_Sn_*x*_ laser fabrication,^1^ but the method cannot offer controlled thickness and Sn content demanding, for example, in quantum well heterostructures.

### Isothermal Growth of Ge_1–*x*_Sn_*x*_ Heterostructures for Electronic and Photonic Device Applications

Using the knowledge gained, different heterostructures have been grown showing the wide possibilities offered by the N_2_ gas flow change method. The aim was to realize Ge_1–*x*_Sn_*x*_ isothermal heterostructures where the Sn content is varied according to the application design requirements. Such heterostructures, discussed below, are illustrated in Table 1. In all cases, the same process temperature of 300 °C, pressure of 60 mbar, and *p*_GeH4_/*p*_SnCl4_ = 1100, as for the reference sample, were used.

**Table 1:** Summary of the Growth Parameters such as Total Carrier Gas (*Q*_T_), Sn Content, Thickness (*d*), and Precursors Flow of the Heterostructures Shown in Figures 3 and 4^a^

		*Q*_T_ (sccm)	Sn content (at. %)	*d* (nm)	SnCl_4_ flow (sscm)	GeH_4_ flow (sscm)
Structure I	top layer	2000	15	60	8	1000
	bottom layer	9000	12	100		
Structure II	top layer	13,000	8	60	6	800
	middle layer	9000	9	75		
	bottom layer	2000	11	90		
Structure III	5 × MQW				6	800
	wells	2000	15.5	30		
	barriers	15,000	9.5	12		
	buffer layer	9000	13	40		
	bottom layer	2000	12	310		

a The “bottom layer” corresponds to the layer grown on the Ge buffer layer while the “top layer” is the last grown layer.

**Figure 3:**
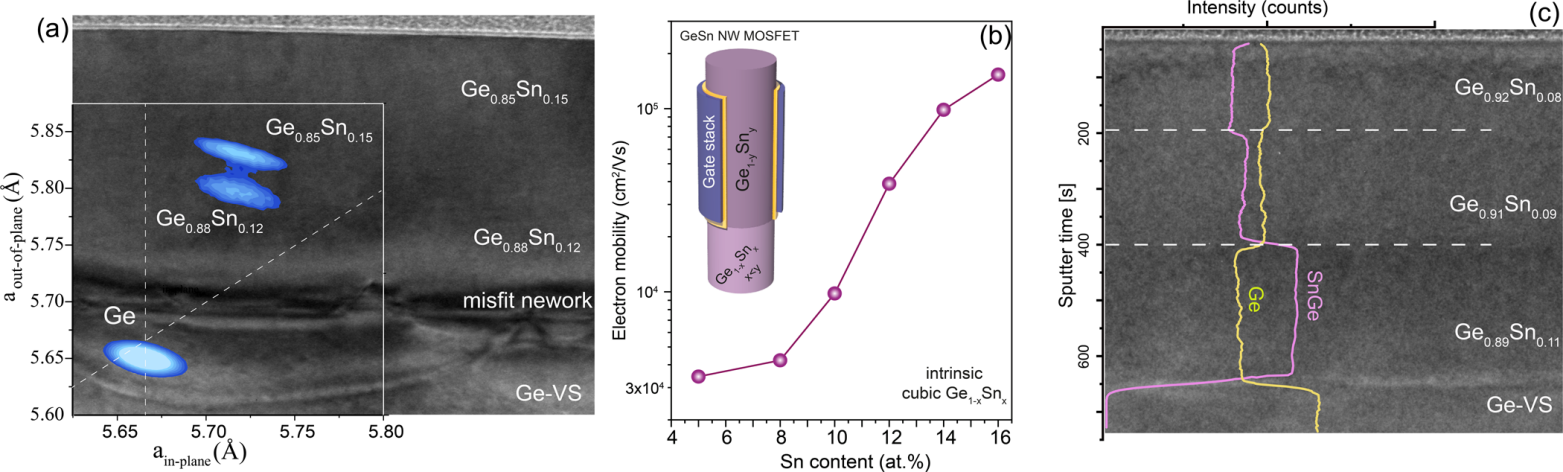
(a) Cross-sectional TEM micrographs of Ge_0.85_Sn_0.15_/Ge_0.88_Sn_0.12_/Ge buffer heterostructure overlapped with the RSM spectrum. (b) Calculated electron mobility vs Sn content. Inset: sketch of a vertical Ge_1–*x*_Sn_*x*_ NW MOSFET. (c) Cross-sectional TEM micrograph of structure II grown at 300 °C varying the N_2_ carrier gas flow, overlapped with SIMS profile of an inverse–step Sn content Ge_0.92_Sn_0.08_/Ge_0.91_Sn_0.09_/Ge_0.89_Sn_0.11_/Ge heterostructure. The Ge intensity is artificially reduced by 50% for better comparison. Some more data are given in the SI, Figures S3 and S4.

Structure I was designed by selecting the optimal values of the Sn and Ge flux that enhance the incorporation of Sn into the alloy (Figure S2 of SI) while the N_2_ flow was decreased from 9000 to 2000 sccm. The N_2_ gas flow was here intentionally continuously changed, leading to a Sn gradient change over ∼40 nm, allowing continuous epitaxial growth without additional strain relaxation as indicated by the RSM spectrum overlapping the TEM micrograph in Figure 3a. In addition, as the thickness of the top layer remains below the critical thickness, the bottom layer is partially relaxed, while the top layer is strained to the first layer. As a result, the first 100 nm-thick layer has 12 at. % of Sn, higher than the sample grown under the “reference” growth conditions with 8.5 at. %. Note that the Ge_0.85_Sn_0.15_ top layer, at *p*_GeH4_ = 300 Pa, is epitaxially grown on Ge_0.88_Sn_0.12_, while Sn segregation appears by direct growth on Ge buffer under similar growth conditions (empty pink symbol in Figure 2a). Structure I design is an upgraded heterostructure for vertical MOSFET devices and CMOS invertors, as recently experimentally demonstrated by Liu et al.^5,40^ The patterning into nanowires (NWs) with diameters below 100 nm results in elastic strain relaxation, offering direct band gap Ge_1–*x*_Sn_*x*_ semiconductors with a large separation between the Γ- and L-valleys. NW MOSFET devices, as sketched in Figure 3b, fully benefit from the high mobility of Γ-electrons in direct band gap Ge_1–*x*_Sn_*x*_ compared to that of L-electrons in the indirect Ge semiconductor, boosting the n-type MOSFET devices. The calculated electron mobility versus Sn concentration for tetragonal and cubic Ge_1–*x*_Sn_*x*_ alloys is presented in Figure 3b. For the electron mobility calculation, the conventional, relaxation time-based calculation, using the 8-band k.p model for Γ-electrons and for holes, and effective-mass (with non-parabolicity) model for L-valley electrons was used.^41−43^

Aiming for ultralow-power electronics, tunneling field-effect transistors (TFETs) were recently investigated.^44,45^ The small effective masses for both holes and electrons^46^ in Ge_1–*x*_Sn_*x*_, alloys and the lower band gap make their use in TFET devices very advantageous. For this purpose, a Ge_1–*x*_Sn_*x*_-based heterostructure design with decreasing Sn content per layer (inverse-step) is proposed in Figure 3c (structure II). The Sn and Ge elemental secondary ion mass spectrometry (SIMS) spectra overlapping a TEM micrograph indicate the Ge_0.89_Sn_0.11_ source (bottom layer) followed by the Ge_0.91_Sn_0.09_ channel (middle layer) and the larger band gap Ge_0.92_Sn_0.08_ drain (top layer). This multilayer structure was isothermally grown at 300 °C, under the “reference” GeH_4_ and SnCl_4_ flux, decreasing the Sn concentration only by increasing the total flow, i.e., *Q*_T_ as indicated in Table 1.

**Figure 4:**
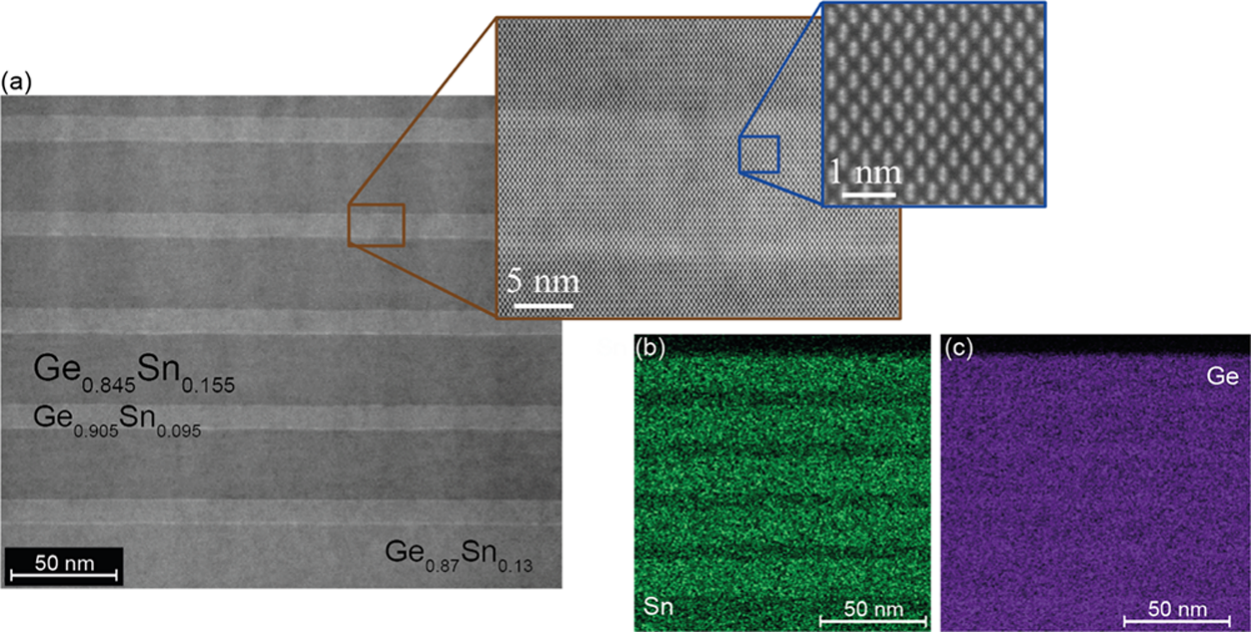
(a) High-resolution TEM and (b, c) energy-dispersive X-ray spectroscopy high-angle annular dark-field (EDS-HAADF) micrographs of a strained Ge_0.845_Sn_0.155_/Ge_0.905_Sn_0.095_ multiple QW heterostructure (structure III). Some more data are given in the SI, Figures S3 and S4.

Finally, the combination of growth methodologies used for structures I and II offer an isothermal multiple quantum well (MQW) heterostructure as shown in structure III (Figure 4). Such a design dramatically reduces the injection current threshold by the carrier confinement, being a pathway for the development of Ge_1–*x*_Sn_*x*_ light sources.^15,47^ Fully strained 5× Ge_0.845_Sn_0.155_/Ge_0.905_Sn_0.095_ QWs were grown on a 40 nm-thick Ge_0.87_Sn_0.13_ buffer layer on an almost relaxed Ge_1–*x*_Sn_*x*_ bottom layer. The TEM/EDS-HAADF micrographs show the very high crystallinity of the epitaxial stack without interface defects between different Ge_1–*x*_Sn_*x*_ layers. Additional data such as XRD diffractograms, RSM scans, SIMS profiles, and HR-TEM micrographs are available in Figures S3 and S4 of the SI.

**Figure 5:**
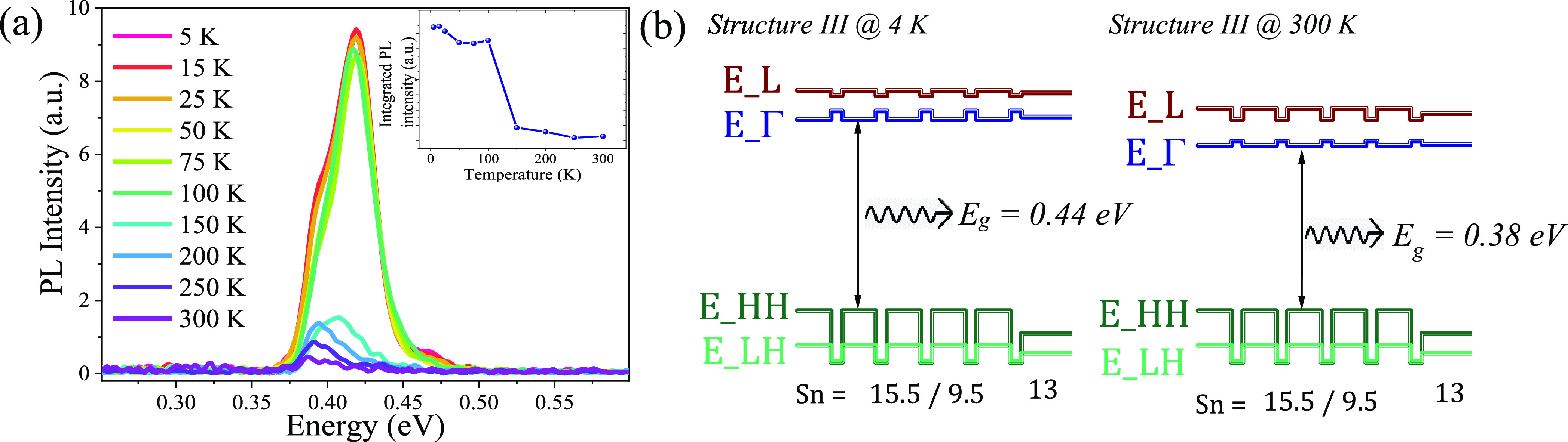
(a) Temperature-dependent PL spectra and (b) electronic band structure calculation at 4 and 300 K for structure III.

The temperature-dependent PL measurements for the structure III are presented in Figure 5a. The PL signal comes from the layers with the lowest band gap energy, here the 15.5 at. % Sn content wells (Table 1). If the temperature decreases, the PL peak position shifts toward higher energy, and the intensity of the PL signal increases, as expected due to the nature of the temperature-dependent band gap.^1^ A clear emission change is visible at 150 K where the intensity decreases abruptly (inset in Figure 5a). If the temperature increases, the conduction band offset between the wells and the barriers reduces and the electrons can escape from the well, resulting in an inefficient confinement effect. Consequently, the MQW behaves similarly to a bulk layer. The PL emission data at both 4 and 300 K are in excellent agreement with the electronic band structure calculation^48^ performed using the Sn and strain profiles determined by RBS and XRD measurements (Figure 5b).

## Conclusions

A simple technical epitaxial growth approach that allows isothermal epitaxy of Ge_1–*x*_Sn_*x*_ semiconductors heterostructures with different compositions has been presented. The simplest method to largely vary Sn incorporation in the Ge lattice is the tuning of the N_2_ flow used as a carrier gas, while keeping the process temperature, deposition pressure, and precursor fluxes constant. Three heterostructures of high-quality Ge_1–*x*_Sn_*x*_ layers were grown with the desired Sn profiles, including the inverse-step Sn content of up to 15 at. % Sn and QW structures targeting different applications such as vertical NW-FETs, tunneling FETs, and mid-IR light emitters. While the growth parameters optimization can be required for specific heterostructure designs, the results presented here prove the suitability of GeH_4_ as a cheaper, stable, and easier-to-handle precursor for state-of-the-art high Sn content Ge_1–*x*_Sn_*x*_ heterostructures.

## Supporting Information Available

Structural characterization of Ge_1–*x*_Sn_*x*_ layers grown at different process temperatures; Sn content and growth rate depending on growth parameters; structural characterization of isothermal heterostructures (PDF)

## Abbreviations

NIR/MIR: near-/mid-infrared
SOC: spin–orbit coupling
QW: quantum well
MOSFET: metal oxide semiconductor field-effect transistor
RP-CVD: reduced-pressure chemical vapor deposition
VS: virtual substrate
RBS: Rutherford backscattering spectroscopy
XRD: X-ray diffraction
RSM: reciprocal space mapping
TEM: transmission electron microscopy
PL: photoluminescence
SI: Supporting Information
NW: nanowire
TFET: tunneling field-effect transistor
SIMS: secondary ion mass spectrometry
MQW: multiple quantum well
EDS-HAADF: energy-dispersive X-ray spectroscopy high-angle annular dark-field
