# Supplementary material for: Isothermal Heteroepitaxy of Ge1–xSnx Structures for Electronic and Photonic Applications
Source: ACS Appl Electron Mater. 2023 Apr 3;5(4):2268–75. doi: 10.1021/acsaelm.3c00112 (PMC10134428; doi:10.1021/acsaelm.3c00112)
Supplement: Supplementary file 1 — el3c00112_si_001.pdf [file el3c00112_si_001.pdf]

Supporting Information

Isothermal heteroepitaxy of $Ge_{1-x}Sn_x$ structures for electronic and photonic applications

Omar Concepción, Nicolaj B. Søgaard, Jin-Hee Bae, Yuji Yamamoto, Andreas T. Tiedemann, Zoran Ikonic, Giovanni Capellini, Qing-Tai Zhao, Detlev Grützmacher, and Dan Buca*

***Omar Concepción** - *Peter Gruenberg Institute 9 (PGI-9), Forschungszentrum Juelich, 52428 Juelich, Germany*; orcid.org/0000-0001-8197-7523; Email: o.diaz@fz-juelich.de

Nicolaj B. Søgaard - *Interdisciplinary Nanoscience Center (iNANO), Aarhus University, 8000 Aarhus C, Denmark*; orcid.org/0000-0002-6718-0910

Jin-Hee Bae - *Peter Gruenberg Institute 9 (PGI-9), Forschungszentrum Juelich, 52428 Juelich, Germany*; orcid.org/0000-0003-0723-9893

Yuji Yamamoto - *IHP - Leibniz Institut für innovative Mikroelektronik, Im Technologiepark 25, 15236 Frankfurt (Oder), Germany*; orcid.org/0000-0003-0928-4356

Andreas T. Tiedemann - *Peter Gruenberg Institute 9 (PGI-9), Forschungszentrum Juelich, 52428 Juelich, Germany*; orcid.org/0000-0001-9194-3554

Zoran Ikonic - *Pollard Institute, School of Electronic and Electrical Engineering, University of Leeds, Leeds LS2 9JT, United Kingdom;* orcid.org/0000-0003-4645-377X

Giovanni Capellini - *IHP - Leibniz Institut für innovative Mikroelektronik, Im Technologiepark 25, 15236 Frankfurt (Oder), Germany*; *Dipartimento di Scienze, Università Roma Tre, Viale G. Marconi 446, 00146, Roma, Italy;* orcid.org/0000-0002-5169-2823

Qing-Tai Zhao - *Peter Gruenberg Institute 9 (PGI-9), Forschungszentrum Juelich, 52428 Juelich, Germany*; orcid.org/0000-0002-2794-2757

Detlev Grützmacher- *Peter Gruenberg Institute 9 (PGI-9), Forschungszentrum Juelich, 52428 Juelich, Germany;* orcid.org/0000-0001-6290-9672

Dan Buca - *Peter Gruenberg Institute 9 (PGI-9), Forschungszentrum Juelich, 52428 Juelich, Germany*; orcid.org/0000-0002-3692-5596

S1. Structural characterization of $Ge_{1-x}Sn_x$ layers grown at different process temperatures.

The classical pathway towards high Sn content layers is the decrease of the process temperature while keeping all other growth parameters constant. Figure S1 shows additional information about the samples presented in Figure 1 of the main text. In the RSM scans of Figure S1a, the shift of the pseudomorphic $Ge_{1-x}Sn_x$ peaks towards lower $a_{out\text{-}of\text{-}plane}$ and closer to the one corresponding to the Ge buffer layer is appreciated as the growth temperature increases. The behavior is more clear with the corresponding nominal values in Figure S1b as well as the residual strain of every layer. With the increase in temperature, the elemental growth rate of Ge exponentially increases while for Sn, the growth rate slightly decreases as the temperature goes up (Figure S1c). This can be an indication that the $Ge_{1-x}Sn_x$ growth kinetics is governed by the Ge growth rate component. Similar behavior was reported by J. Aubin et al.[1] using Ge_2H_6 and $SnCl_4$ as precursors. The activation energy of 0.24 eV was determined through the Arrhenius plot of Figure S1d. This value is lower than the ones previously reported by our group between 0.4 – 0.7 eV[2,3] but using Ge_2H_6 as precursor flow. Result that is in line with J. M. Hartmann et al. findings[4].

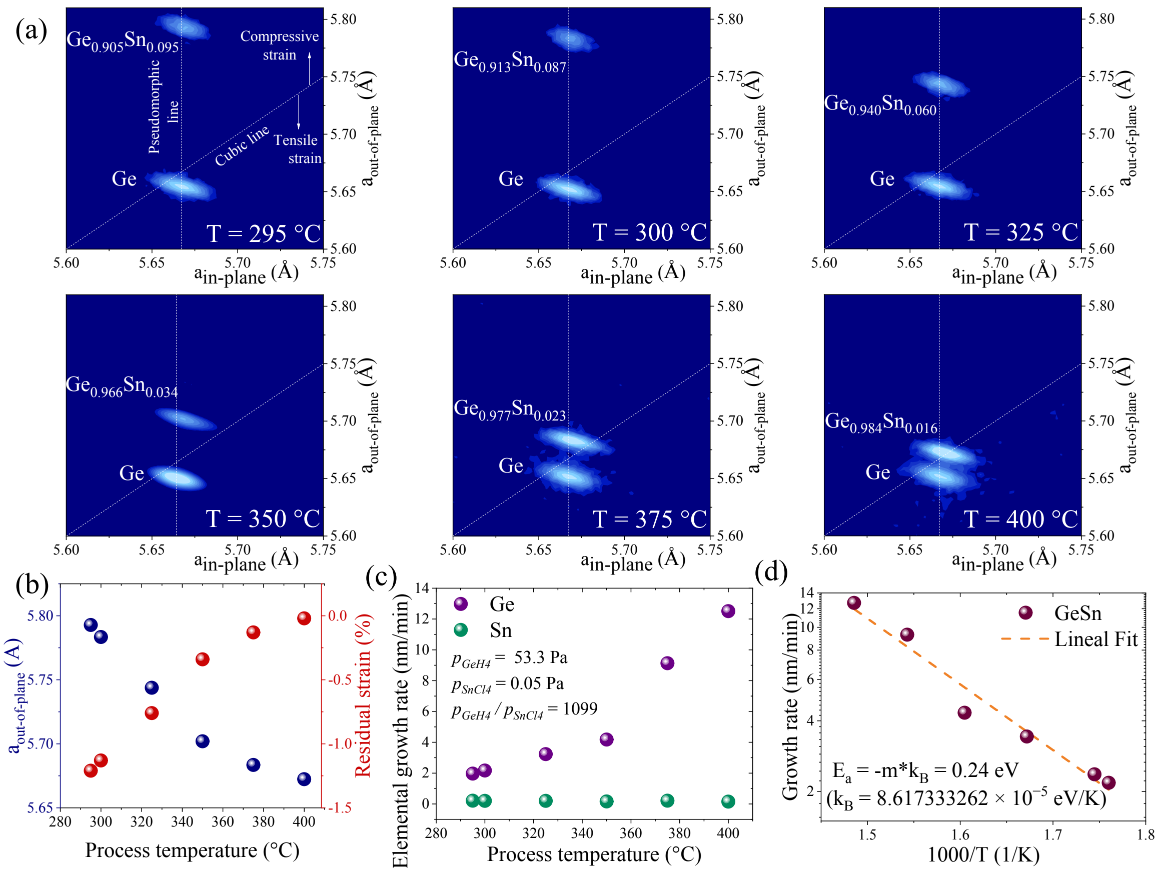

Figure S1: *(a) Asymmetric RSM scans along the (224) plane of $Ge_{1-x}Sn_x$ layers grown at different process temperatures. The dotted line corresponds with the pseudomorphic and cubic lines. (b) Out-of-plane lattice constant and the residual strain of the same set of $Ge_{1-x}Sn_x$ layers. (c) Growth rate of Ge and Sn elements. (d) Arrhenius plot for the $Ge_{1-x}Sn_x$ growth rate.*

S2. Sn content and growth rate depending on growth parameters.

In this Section, a detailed description of the influence of GeH_4 flow, $SnCl_4$ flow, and reactor pressure in the incorporation of Sn in the alloy is given. In all the cases, with the exception of the parameter under study, the rest remained constant and equal to those corresponding to the reference sample: partial pressure of GeH_4 (p_{GeH4} = *53.33 Pa*), partial

pressure of $SnCl_4$ (p_{SnCl4} = 0.05 Pa), reactor pressure (P_{react} = 60 mbar), total gas flow (Q_T = 9000 sccm), and growth temperature (T = 300˚C).

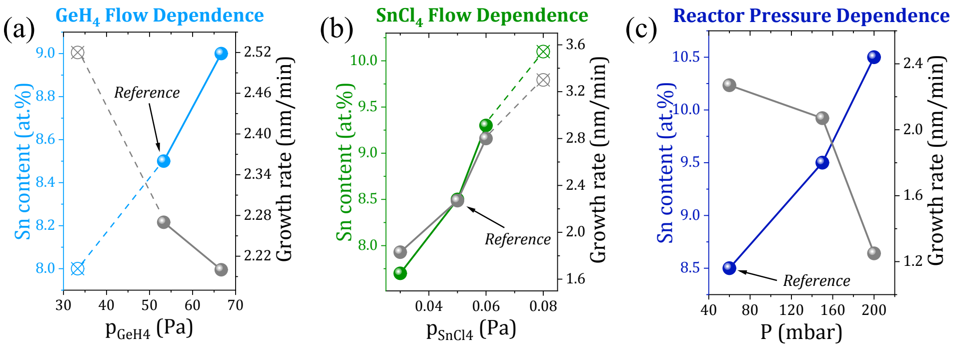

(d)

	GeH₄ Flow Dependence	*SnCl₄ Flow Dependence*	*Reactor Pressure Dependence*
Temperature (°C)	300	300	300
GeH_4 Flow (sccm)	**500 – 1000**	800	800
p_{GeH4} (Pa)	33.3 – 66.7	53.3	53.3 – 177.8
$SnCl_4$ Flow (sccm)	6	**4 – 10**	6
p_{SnCl4} (Pa)	0.05	0.03 – 0.08	0.05 – 0.16
p_{GeH4}/p_{SnCl4}	684-1374	1648 – 659	1099
Total Flow (sccm)	9000	9000	9000
Pressure (Pa)	60	60	**60 – 200**

Figure S2: *Sn content and growth rate depending on (a) GeH_4 flow, (b) $SnCl_4$ flow, and (c) reactor pressure of different $Ge_{1-x}Sn_x$ layers grown at 300°C. The empty symbols with a cross represent samples with a Sn segregation appearance on the surface. The "reference" growth conditions are p_{GeH4} = 53.3 Pa, p_{SnCl4} = 0.05 Pa, reactor pressure of P_{react} = 60 mbar, and total gas flow Q_{Total} = 9000 sccm. (d) The Table shows the growth parameters of every sample showed in (a-c).*

Starting from the reference growth conditions, the decrease/increase in the GeH_4 and $SnCl_4$ partial pressures through the direct control of the flux of these precursors (Figures S2a-b) can be made within very narrow ranges, due to technical limitations of the CVD reactor. This results in small changes of the Sn incorporation, giving a maximum Sn concentration of

9.0 – 9.5 at.%, compared to the 8.5 at.% Sn obtained under the reference growth conditions. Figure S2c shows the Sn content dependence with the reactor pressure. A change between 60 and 200 mbar translates into a $SnCl_4$ partial pressure between 0.05 Pa (reference growth conditions) and 0.16 Pa. The maximum Sn incorporation of 10.5 at.% is obtained at the highest reactor pressure of 200 mbar (p_{SnCl4} =0.16 Pa).

S3. Structural characterization of isothermal heterostructures

The characterization of the *Structures* presented in the main text is shown in this section (Figure S3-4). The crystal structure study is presented by the XRD and RSM results. SIMS shows the composition profile through the sample and TEM the interfaces and atomic arranges.

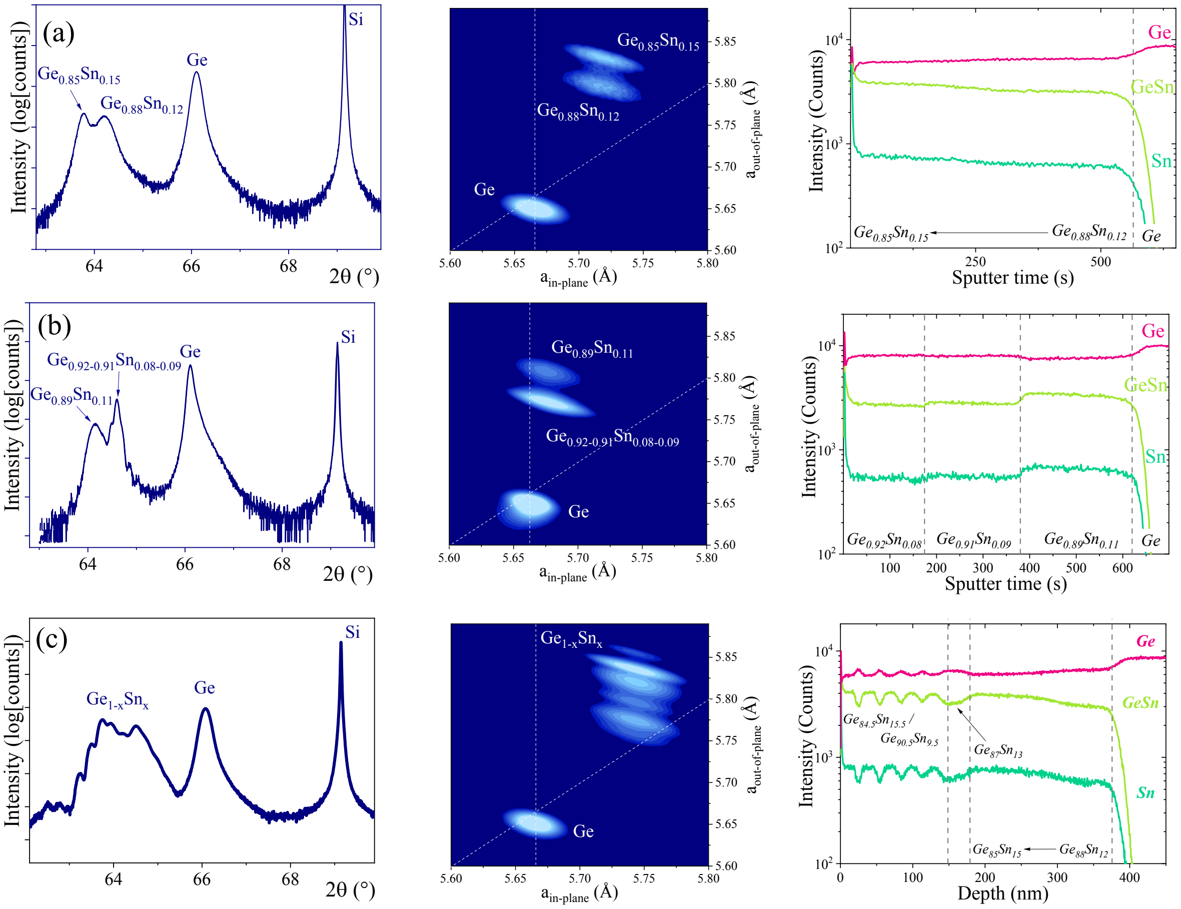

Figure S3: *Symmetric 2θ-ω XRD spectra along the (004) plane, asymmetric RSM scans along the (224) plane, and SIMS profiles of Structure I (a), Structure II (b), and Structure III (c), respectively.*

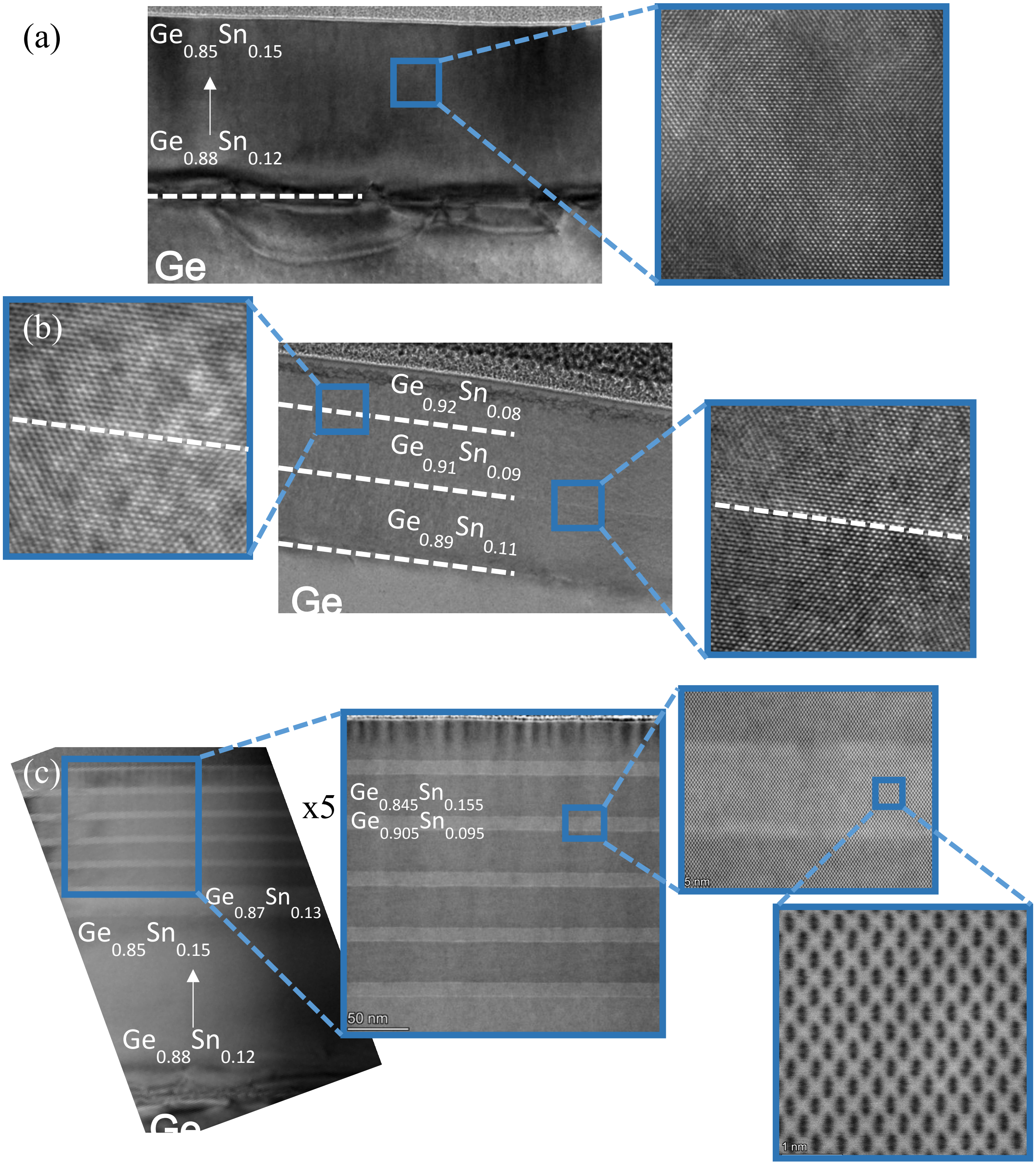

Figure S4: *Cross-sectional high-resolution TEM micrographs of Structure I (a), Structure II (b), and Structure III (c), respectively.*

References

(1) Aubin, J.; Hartmann, J. M. GeSn Growth Kinetics in Reduced Pressure Chemical Vapor Deposition from Ge2H6 and SnCl4. *J. Cryst. Growth* **2018**, *482*, 30–35. https://doi.org/10.1016/j.jcrysgro.2017.10.030.

(2) Wirths, S.; Buca, D.; Tiedemann, A. T.; Holländer, B.; Bernardy, P.; Stoica, T.; Grützmacher, D.; Mantl, S. Epitaxial Growth of Ge 1-x Sn x by Reduced Pressure CVD Using SnCl 4 and Ge 2 H 6. *ECS Trans.* **2013**, *50* (9), 885–893. https://doi.org/10.1149/05009.0885ECST/XML.

(3) Von Den Driesch, N.; Stange, D.; Wirths, S.; Mussler, G.; Holländer, B.; Ikonic, Z.; Hartmann, J. M.; Stoica, T.; Mantl, S.; Grützmacher, D.; Buca, D. Direct Bandgap Group IV Epitaxy on Si for Laser Applications. *Chem. Mater.* **2015**, *27* (13), 4693–4702. https://doi.org/10.1021/ACS.CHEMMATER.5B01327.

(4) Hartmann, J.-M.; Frauenrath, M.; Richy, J. Epitaxy of Pseudomorphic GeSn Layers with Germane (GeH 4) or Digermane (Ge 2 H 6) as Ge Precursors and Tin Tetrachloride (SnCl 4) as the Sn Precursor. *ECS Trans.* **2020**, *98* (5), 225–238. https://doi.org/10.1149/09805.0225ecst.
